# Effect of the Electromagnetic Field (EMF) Radiation on Transcriptomic Profile of Pig Myometrium during the Peri-Implantation Period—An In Vitro Study

**DOI:** 10.3390/ijms22147322

**Published:** 2021-07-07

**Authors:** Ewa Monika Drzewiecka, Wiktoria Kozlowska, Lukasz Paukszto, Agata Zmijewska, Pawel Jozef Wydorski, Jan Pawel Jastrzebski, Anita Franczak

**Affiliations:** 1 Department of Animal Anatomy and Physiology, Faculty of Biology and Biotechnology, University of Warmia and Mazury in Olsztyn, 10-719 Olsztyn, Poland; ewa.drzewiecka@uwm.edu.pl (E.M.D.); kozlowskawiktoria@outlook.com (W.K.); agata.zmijewska@uwm.edu.pl (A.Z.); pawel.wydorski@uwm.edu.pl (P.J.W.); 2 Department of Plant Physiology, Genetics and Biotechnology, Faculty of Biology and Biotechnology, University of Warmia and Mazury in Olsztyn, 10-719 Olsztyn, Poland; lukasz.paukszto@uwm.edu.pl (L.P.); jan.jastrzebski@uwm.edu.pl (J.P.J.)

**Keywords:** electromagnetic field, next-generation sequencing, RNA editing sites, lncRNA, myometrium, pig

## Abstract

The electromagnetic field (EMF) affects the physiological processes in mammals, but the molecular background of the observed alterations remains not well established. In this study was tested the effect of short duration (2 h) of the EMF treatment (50 Hz, 8 mT) on global transcriptomic alterations in the myometrium of pigs during the peri-implantation period using next-generation sequencing. As a result, the EMF treatment affected the expression of 215 transcript active regions (TARs), and among them, the assigned gene protein-coding biotype possessed 90 ones (differentially expressed genes, DEGs), categorized mostly to gene ontology terms connected with defense and immune responses, and secretion and export. Evaluated DEGs enrich the KEGG *TNF signaling pathway*, and *regulation of IFNA signaling* and *interferon-alpha/beta signaling* REACTOME pathways. There were evaluated 12 differentially expressed long non-coding RNAs (DE-lnc-RNAs) and 182 predicted single nucleotide variants (SNVs) substitutions within RNA editing sites. In conclusion, the EMF treatment in the myometrium collected during the peri-implantation period affects the expression of genes involved in defense and immune responses. The study also gives new insight into the mechanisms of the EMF action in the regulation of the transcriptomic profile through lnc-RNAs and SNVs.

## 1. Introduction

The electromagnetic field (EMF) term stands for the electromagnetic energy generated by geological structures in the Earth’s shell and by all the electric-powered devices. Currently, the EMF is recognized as a physical pollutant of the environment that may jeopardize human health [1,2,3,4,5]. In 1996, the World Health Organization started “The International EMF Project”, which aims to establish and assess the health and environmental effects of exposure to static and time-varying EMFs. The most common power frequencies of the EMF range from 50 to 60 Hz, which are classified as extremely low frequencies of the EMF (ELF-EMF) [5]. The number of ELF-EMF sources in everyday life is relatively high, as they are generated by the vast majority of household devices, lighting, and heating [1,3]. Thus, the consequences of the ELF-EMF exposition on the living organisms need to be elucidated.

Previous studies provided shreds of evidence that the exposition to the EMFs affects the reproductive processes in mammals [2,4,6,7,8]. In females, the EMF radiation altered the course and length of the estrous cycle, ovulation and conception rate, the dynamics of fetal growth, and pregnancy outcome [2,4,6]. Nevertheless, the molecular background of these changes is hardly understood. Due to the fact that the success of early pregnancy is ensured by a unique embryo-maternal dialog involving the action of steroid hormones [9,10], it was proposed that jeopardizing effects of the EMF treatment on pregnancy development might be coupled with the altered uterine steroidogenic activity [7,8,11,12]. Indeed, recent in vitro studies on a pig model demonstrated that the EMF treatment (50 and 120 Hz, 8 mT, 2 and 4 h) induces alterations in the synthesis and secretion of steroid hormones by the uterine tissues, including the myometrium [7,8,11,12]. Notably, in pigs, the myometrium contributes significantly to the total uterine production of androstenedione (A_4_) [13], which induces anabolic processes in the target tissues and regulates morphogenesis, cellular proliferation, and hyperplasia [14]. Therefore, an observed decrease in A_4_ production by myometrial tissue exposed to an EMF [8] may decrease the myometrial potential for structural remodeling required during pregnancy. Importantly, embryo-maternal dialog during the early stages of pregnancy involves elements of the immune system and the action of cytokines, gonadotropins, and growth factors [15,16,17]. It was established that cytokines, gonadotropins, and growth factors affect myometrial activity [18,19,20,21]. Thus, it is interesting whether EMF treatment may affect immunological processes in the tissue, possibly affecting the course of the implantation process.

Providing the above, for better understanding of the EMF-related effects on reproductive processes, we aimed to determine global transcriptomic alterations in the myometrium treated in vitro with an EMF. We hypothesized that in response to the EMF treatment the transcriptomic activity of the myometrium changes and these alterations may impair proper course of molecular events, including embryo–maternal cross talk, during the peri-implantation period. Hence, in the current study the myometrial explants collected from pigs during the fetal peri-implantation period were exposed in vitro for 2 h to the EMF at a frequency of 50 Hz, which is classified to widely occurring ELF-EMFs in human everyday life [3], and with a magnetic induction of 8 mT, which is a typical exposure level from EMF sources for industry and therapeutic equipment used in human medicine [22] for further examination of the tissue transcriptomic profile. To obtain a wide view of possible EMF-related changes in the transcriptomic activity of the tissue and provide an insight into the possible mechanism of these changes, the identification of differentially expressed genes (DEGs) and differentially expressed non-coding RNAs (DE-ncRNAs), and prediction of possible RNA editing sites in the myometrium exposed to EMF radiation were provided. The obtained results contribute to a better understanding of the molecular background of the alterations in the tissue evoked by exposition in vitro to the EMF treatment.

## 2. Results

### 2.1. The Statistics of RNA Sequencing

**Figure 1:**
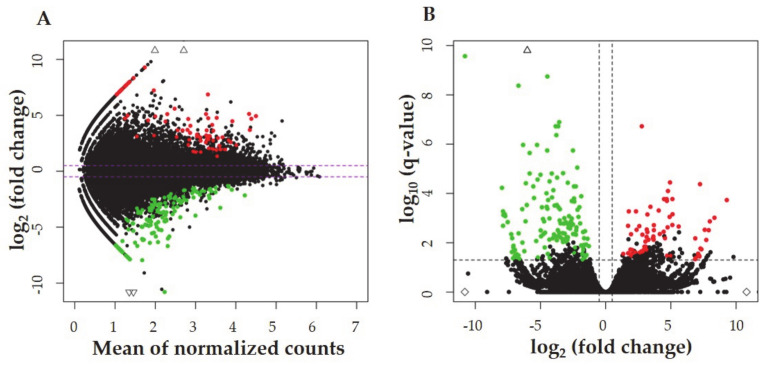
(**A**) MA plot depicts the logarithmic scale of the fold changes (logFC) in the *Y*–axis, and the count mean expression in the *X*–axis. (**B**) The volcano plot describes logFC in the *X*–axis and logarithmic adjusted *p*-value in the *Y*–axis. Red dots illustrate upregulated transcriptionally active regions (TARs), green dots represent downregulated TARs.

The overall statistics of RNA-seq data were constructed for six cDNA libraries (three extracted from myometrium treated with the EMF, and three extracted from the controls). After sequencing, 296,791,628 raw paired reads were obtained, on average 49.47 mln per sample. The 284,845,072 filtered reads were mapped to the pig genome (version Ss11.1.98), and the unique mapped rate ranged between 77.39% and 93.71%. The distribution of mapped reads to genes’ structures was: 51% of reading pairs mapped to coding sequences, 8% mapped to introns, 27% aligned to untranslated regions, and the remaining 14% mapped to intergenic regions. An overview of changes in gene expression is illustrated in MA and volcano plots (Figure 1A,B). PCA plot and hierarchical clustering of RNA-seq results are presented in Figure S1.

### 2.2. The Identification of Differentially Expressed Genes (DEGs), and Differentially Expressed Non-Coding RNAs (DE-ncRNAs)

**Figure 2:**
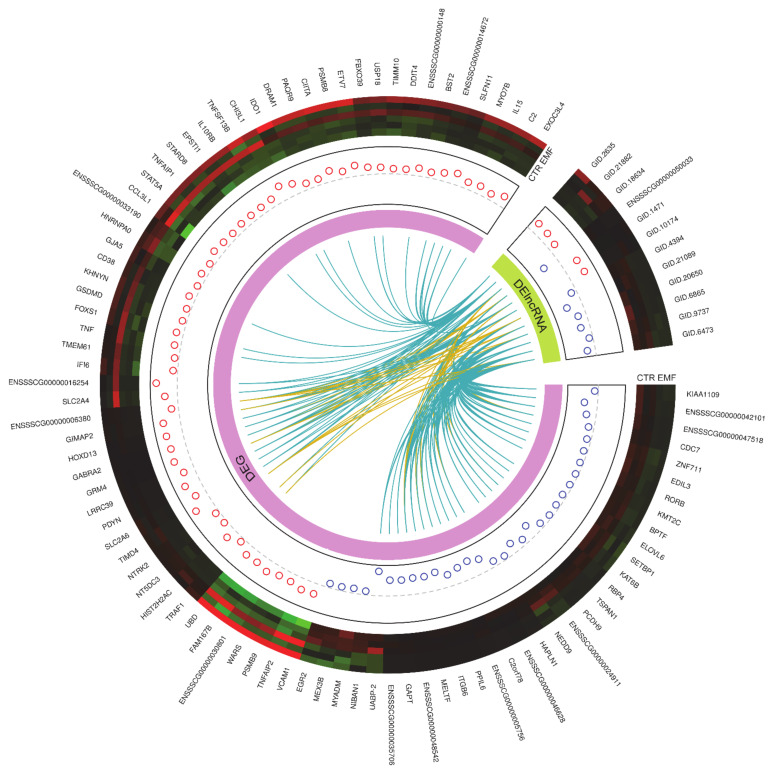
Visualization of differentially expressed genes (DEGs) and long noncoding RNAs (DE-lncRNAs) statistically significant (P-adjust < 0.05) in the impact of the electromagnetic field. The external circle represents the gene ID (gene names, ENSEMBL ID, StringTie ID) of DEGs (large part) and DE-lncRNAs (small part). Six upper tracks depict the normalized (Z-score; red-green scale) expression values for DEGs and DE-lncRNAs in each RNA-seq library. The circles in the next track describe increased (red) and decreased (blue) expression (logFC) in the compared groups. The next track shows the links between the correlated DEGs and DE-lncRNAs, where blue links depict positive correlation and yellow negative. Figure created with circlize Bioconductor library and custom R script.

The analysis of DEGs was performed at a false discovery rate (FDR) < 0.05%. A 2 h treatment duration with the EMF at 50 Hz affected the expression of 215 differentially expressed transcriptionally active regions (DE-TARs), among which there were 90 protein-coding genes and 12 differentially expressed long non-coding RNAs (DE-lncRNAs) (Figure 2). The others were represented by non-classified DE-ncRNAs. Four out of the identified DE-lncRNAs were annotated in the ENSEMBL database. The majority of the evaluated DE-TARs, i.e., 67.44% was down-regulated. Among the down-regulated TARs, the assigned protein-coding annotation have 32 out of 145 transcripts, and among the up-regulated TARs the assigned ENSEMBL gene names have 58 out of 70 DE-TARs candidates. The DE-TARs with the assigned gene protein-coding biotype were described as DEGs. The top five up-regulated DEGs in response to the EMF treatment i.e., DEGs with the highest log2FC values are ENSSSCG00000016254, *GTPase, IMAP family member 2* (*GIMAP2*), *glutamate metabotropic receptor 4* (*GRM4*), *homeobox D13* (*HOXD13*), *T cell immunoglobulin and mucin domain-containing 4* (*TIMD4*), whereas the top five down-regulated DEGs, i.e., DEGs with the lowest log2FC values are: *uteroferrin associated basic protein-2* (*UABP-2*), *chromosome 2 open reading frame 78* (*C2orf78*), *integrin subunit beta 6* (*ITGB6*), ENSSSCG00000048542, and *melanotransferrin* (*MELTF*). A full list of the evaluated DE-TARs in porcine myometrium treated with the EMF at 50 Hz for 2 h can be found in Table S1.

For the 125 DE-ncRNAs, a filtration procedure was applied for the elimination of protein-coding genes, short sequences, one-exon transcripts, and sequences with high coding potential. As a result, 12 transcripts indicated as DE-lncRNAs were obtained. Among them, one transcript (ENSSSCG00000050033) was classified in the ENSEMBL database as lncRNA biotype, the others were novel, identified in our research. Transcription regulatory machinery when EMF was imposed on the porcine myometrium revealed five up-regulated and seven down-regulated DE-lncRNAs. All DE-lncRNAs showed trans-interaction with 55 target genes, due to the similarity of expression profiles (absolute value of the Pearson correlation coefficient > 0.7).

### 2.3. Functional Ontology Annotations

**Figure 3:**
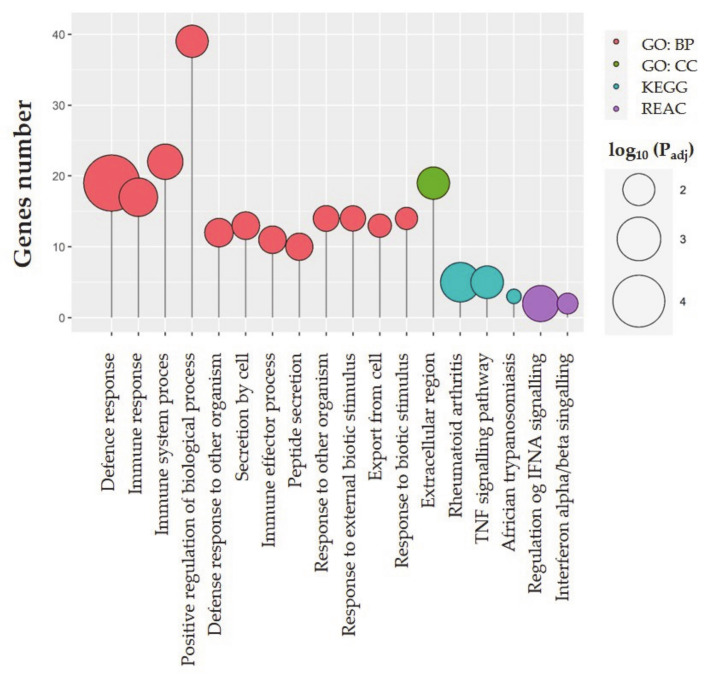
Lollipop chart of the ontology terms (biological process, cellular components, metabolic and signaling pathways) detected during Gene Ontology (GO) and KEGG analysis. Circle size refers to the logarithmic scale of adjusted *p*-value in enrichment GO analysis. *X*-axis described the number of DEGs involved in enriched GO terms.

The total number of 76 DEGs were assigned to the functional ontology annotations visualized in Figure 3. There were evaluated 12 gene ontology (GO) annotations for biological processes (BPs) terms, and one cellular component (CC) term—extracellular region. Apart from the evaluated GO BP positive regulation of biological process (GO:0048518), eight of the evaluated GO BPs relate to defense and immune responses to different factors, and three of them are connected with secretion and export. There were evaluated three KEGG pathways, i.a. *TNF signaling pathway* (KEGG: 04668) and two REACTOME pathways, namely *regulation of IFNA signaling* (REAC: R-SSC-912694), and *interferon-alpha/beta signaling* (REAC: R-SSC-909733). The evaluated GO terms and KEGG and REACTOME pathways are presented in Table 1, and the full list of evaluated GO terms, KEGG and REACTOME pathways with full statistical parameters and assigned intersections can be found in Table S2.

### 2.4. RNA Editing Site Prediction

**Figure 4:**
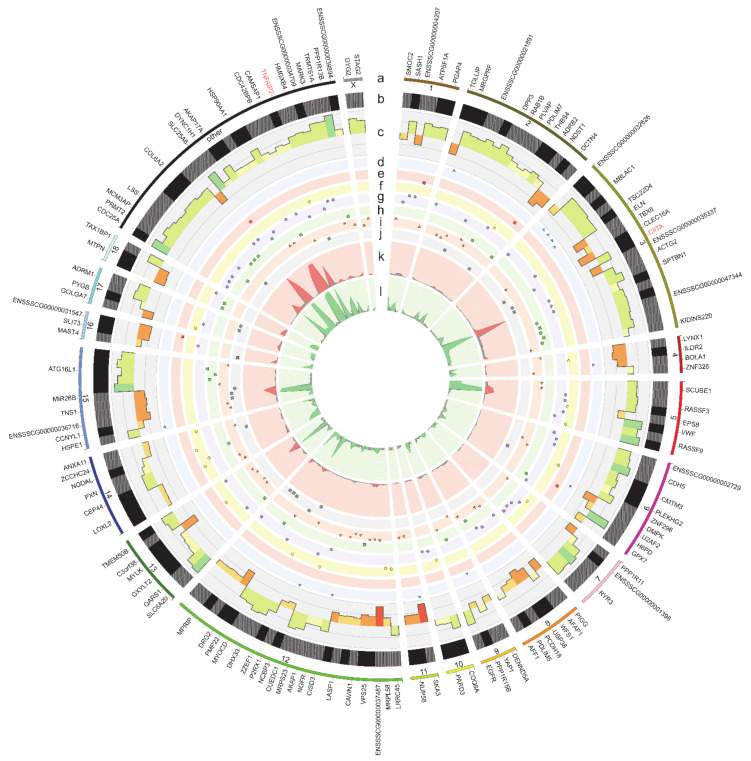
Visualization of the RNA editing candidates exposed to the EMF. (**a**) The external circle represents the porcine chromosomes and other unclassified scaffolds, where the region length is proportional to the number of editing substitutions. Gene names (DEGs are marked in red) identified in the vicinity of RNA candidates are placed outside the track. (**b**) The second track (black and grey lined blocks) presents an abundance of adenine to inosine and cytosine to thymine (A-to-I and C-to-T) types of RNA editing sites. (**c**) The third track, the histogram, depicts the difference in the alternative allele fraction (ΔAAF) between the EMF-treated samples and the controls. (**d**–**j**) The next seven middle scatter plots present the localization of RNA editing substitutions on upstream (blue triangles), 5’UTR (red rectangle), missense (yellow circle), synonymous (purple circle), intron (green squares) 3’ UTR (orange triangle), and downstream region (grey rectangle); vertical axis on each scatter plot shows the normalized FDR. (**k**,**l**) The two inner tracks show the coverage of the alternative (red histogram) and reference variants (green histogram) in all the RNA-seq libraries. Figure created with Circos software.

Variant calling analysis revealed 195,361 single nucleotide variants (SNVs) in the myometrial tissue treated in vitro with the EMF at 50 Hz. Using high-quality Genome Analysis Tool Kit (GATK) filters 36,189 SNVs were removed from the downstream analysis. Screening the porcine genome 74,475 substitutions located near to bidirectional genes, simple sequence repeats, paralogs, and splice junction regions were filtered out. Investigating the variant calling frequency were obtained 40,123 polymorphic sites with an alternative allele in at least half of RNA-seq libraries. Next, 37,748 substitutions annotated as single nucleotide polymorphisms (SNPs) and possessing too high levels of alternative alleles frequency (AAF > 0.7) in any experimental sample were removed. After the filtering procedure, 527 substitutions showed a significant (FDR < 0.001, ∆AAF > 0.1 and <−0.1) imbalance in the expression of alternate allele between the EMF-treated and the control samples. Finally, after a variant annotation procedure, 182 candidates were assigned as RNA canonical substitutions (A to I and C to T) in the porcine myometrial transcriptome (Figure 4; Table S3). The VEP assigned RNA editing candidates to the following main variation consequences: 31—upstream gene, 17—5’UTR, 22—missense, 60—synonymous, 35—intronic, 65—3’UTR, and 50—downstream variants. Twelve out of the identified RNA editing variants were found within downstream gene variants of *class II major histocompatibility complex transactivator* (*CIITA*) and *TNF alpha-induced protein 2* (*TNFAIP2*) (Figure 4). Additionally, the substitution localized within an intron of protein phosphatase 1 regulatory subunit 15B (PPP1R15B) was assigned to the Sus scrofa PRE-1 SINE region.

### 2.5. Validation of NGS Results

**Figure 5:**
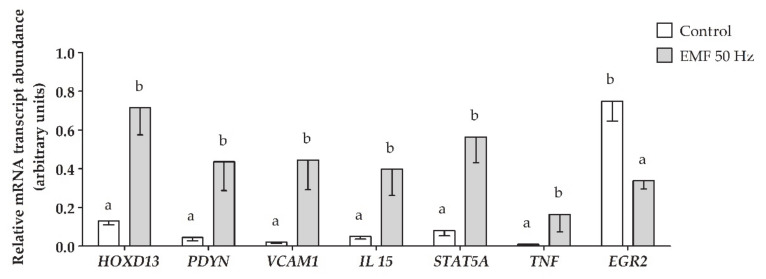
The relative mRNA expression of genes with the altered expression in porcine myometrium during the peri-implantation period, in response to the EMF treatment (50 Hz, 2 h, 8 mT). mRNA transcript abundance values are presented as men 2^−∆∆Ct^ values ± SEM. Different lower-case letters (a, b) indicate statistically significant differences in gene expression between control (no EMF) and experimental (EMF-treated) samples.

In the myometrial tissue treated in vitro with an EMF at 50 Hz when compared to the control the relative mRNA transcript abundance of *homeobox D13* (*HOXD13*), *prodynorphin* (*PDYN*), *vascular cell adhesion molecule 1* (*VCAM1*), *interleukin 15* (*IL15*), *signal transducer and activator of transcription 5A* (*STAT5A*), and *tumor necrosis factor α* (*TNF*) was significantly increased, whereas the relative mRNA transcript abundance of *early growth response protein 2* (*EGR2*) was significantly decreased. The direction of these changes (up- or downregulation) correspond with the results obtained using NGS analysis. Thus, the validation procedure confirmed the consistent and high quality of the obtained NGS results. The expression profiles of mRNAs encoded by validated genes are presented in Figure 5.

## 3. Discussion

The results from the present in vitro study documented for the first time the alterations occurring in the transcriptome of the myometrium in the consequence of the EMF radiation at a frequency of 50 Hz after a short duration (2 h) of in vitro treatment. Specifically, the EMF treatment affected the expression of 223 TARs, and the majority of them, i.e., 65.47% was down-regulated. Notably, ~36% of the DE-TARs were classified as DEGs, and the remaining ones were classified as DE-ncRNAs, including DE-lncRNA, and other unclassified DE-ncRNA. Most of the evaluated DEGs were up-regulated and categorized to GO BPs terms connected with defense and immune responses to different factors, as well as to GO BPs terms related to the secretion and export. Moreover, the evaluated DEGs enrich the KEGG *TNF signaling pathway* and *regulation of IFNA signaling* and *interferon-alpha/beta signaling* REACTOME pathways. Thus, the current in vitro study shows that EMF action may concern the modulation of immune-dependent processes in the myometrium. Notably, it was found that the EMF treatment can evoke RNA editing events. Three out of all SNVs occurring in the myometrium in response to the EMF occur within (missense variant) and/or in the vicinity (downstream gene variant) of two evaluated DEGs, i.e., *class II major histocompatibility complex transactivator* (*CIITA*), and *TNF alpha-induced protein 2* (*TNFAIP2*). All of these alterations give some insight into the impact of EMF radiation on the regulation of gene expression in the myometrium.

This study determined that in vitro treatment with an EMF at a frequency of 50 Hz within 2 h of treatment duration alters the myometrial expression of genes enriching *defense response* (GO:0006952), *immune response* (GO:0006955), *immune system process* (GO:0002376), and *immune effector process* (GO:0002252) GO BPs terms. Common for all of these terms were up-regulated *C-C motif chemokine ligand 3 like 1* (*CCL3L1*, FC = 3.696), *interleukin 15* (*IL15*), FC = 3.659, *signaling lymphocytic activation molecule family member 1* (*SLAMF1*) (ENSSSCG00000006380, FC = 3.124), *tumor necrosis factor* (*TNF*, FC = 2.812), *bone marrow stromal cell antigen 2* (*BST2*, FC = 2.579), and *interferon-alpha inducible protein 6* (*IFI6*, FC = 2.043) genes. These genes encode for proteins known for their proinflammatory action (CCL3L1, SLAMF1) [23,24,25,26,27,28,29] and immunomodulatory function (IL 15, TNF) [30,31,32,33], or act as an exosomal tether (BST2) [34] or proliferative and anti-apoptotic factors (IL15, IFI6) [35,36,37]. Recent studies have shown that in general, a short-lasting ELF-EMF treatment (2–24 h/day, up to a week) increases immune responses, while long-term-lasting ELF-EMF treatment (2–24 h/day up to 8 years) contributes mainly to the immunosuppression [38]. Moreover, ELF-EMF treatment was found to exert immunomodulatory effects, especially in the case of innate immunity [39,40]. Notably, the EMF therapies were defined as safe and effective in pain and/or inflammation therapies [41]. Thus, the results of the current study may contribute to the understanding of the molecular background of the consequences of exposure to the ELF-EMF stimulation in everyday life or healing with the usage of the ELF-EMF radiation.

The present study evaluated that the ELF-EMF treatment induces alterations in the *regulation of IFNA signaling* (REAC: R-SSC-912694) and *interferon-alpha/beta signaling* (REAC: R-SSC-909733) REACTOME pathways. These pathways are enriched by the up-regulated in the myometrium *interleukin 10 receptor subunit beta* (*IL10RB*) and *ubiquitin-specific peptidase 18* (*USP18*) genes. Interferons and IL 10 are important activators of the Janus kinases-signal transducer and activator of transcription proteins (JAK-STAT) signaling pathway, which may i.a. modulate the expression of genes coding for cytokine receptors [42]. The current study determined that myometrial *STAT5A* mRNA transcript abundance was significantly increased in response to the ELF-EMF treatment. Studies on a mouse model revealed that the expression of *STAT5A* is required for normal proliferation and development of T and natural killer (NK) cells, and determines the synthesis of IL 2 and IL 15 [42]. Interestingly, it was found that the ELF-EMF treatment at a frequency of 60 Hz increases phosphorylation and therefore, the activation of STAT5 in mouse Th cells [43]. Possibly, the observed up-regulation of *STAT5A* transcriptional activity in the myometrium in the response to the ELF-EMF treatment corresponds to its activation via phosphorylation, which is likely to trigger the stimulation of immune processes in myometrial tissue by regulating the expression of immunostimulatory and immunomodulatory factors.

Notably, the present study revealed that in vitro EMF treatment causes an up-regulation of *IL15* gene transcriptional activity. IL 15 is a pleiotropic cytokine playing a role in protective immune response, immunosuppression, allograft rejection, and the pathogenesis of autoimmune diseases, modulates immune responses against intracellular pathogens, functions as a growth factor, myokine, and repressor of apoptotic events [30,33]. Moreover, IL 15 is an essential growth factor for uterine NK cells, which together with macrophages infiltrate the uterus especially during early pregnancy acting positively on the implantation [15,44]. Importantly, uterine NK cells and macrophages produce a variety of cytokines that support embryo-maternal interactions and implantation [15,44]. Thus, a greater myometrial expression of *IL15* observed in the consequence of in vitro treatment with an EMF at a frequency of 50 Hz may contribute to the creation of an immunological condition that may support the process of implantation. Nevertheless, further in vivo studies are needed for deeper insight into the relation between EMF treatment, immune responses, and implantation.

IL 15 is a part of the *TNF signaling pathway* (KEGG: 04668), which, in the current study, was documented to be affected in the myometrium as the consequence of the ELF-EMF treatment. Interestingly, it is the first report documenting the myometrial expression of *TNF*, while the expression of *TNF receptor 1* (*TNFRI*) mRNA in this tissue has been already determined [19]. In the myometrium, TNF stimulates estradiol-17*β* (E_2_) and prostaglandin E_2_ (PGE_2_) release by the myometrium of gravid pigs on days 15–16 [19] and day 40 [45] of pregnancy, respectively. The effect of the EMF treatment on myometrial prostaglandins synthesis has not been studied yet; however, its stimulating effect on estrogen production in the tissue has been confirmed [7]. Possibly, there is a relation between the EMF-stimulated increase of *TNF* mRNA transcript abundance and E_2_ release.

Notably, TNF induces the transcriptional activation of *TNFAIP2*, which is considered as a primary response gene to TNF action [46]. The results from the present study provided shreds of evidence that in response to the EMF treatment *TNFAIP2* expression in the myometrium was up-regulated, and two canonical RNA editing candidates in regions 1,813,104 and 1,818,701 of *TNFAIP2* were identified. Alterations in *TNFAIP2* are frequently coupled with human diseases, including cancers and infectious diseases [47]. Notably, the treatment of the myometrium with TNF increases the transcriptional activity of activin A, a pro-fibrotic marker highly expressed in uterine leiomyomas [48,49,50,51]. One may not exclude that the observed transcriptomic alterations in myometrial *TNF* and *TNFAIP2*, occurring in response to the ELF-EMF treatment may cause a neoplastic lesion in the tissue. On the contrary, studies using the HaCaT keratinocyte cells model determined that the EMF treatment at a frequency of 50 Hz modulates the expression and release of TNF in damaged cells which helps in wound healing [52]. It cannot be discounted that the final consequences of the interaction among the ELF-EMF treatment, the expression, release, and molecular and physiological effects of TNF action may be strictly related to the type of tissue exposed to EMF and needs further in vivo investigation.

Interestingly, the *TNF signaling pathway* (KEGG: 04668) is enriched also by an up-regulated in the current study *VCAM1* gene. The expression of *VCAM1* is stimulated by proinflammatory cytokines, including TNF [53]. Thus, there may occur a genetic interaction between *VCAM1* and *TNF*. During inflammation, VCAM1 enables the adhesion of leukocytes to the endothelium and enables their transmigration [54]. VCAM1 also stimulates angiogenesis and increases cell-cell contact and interactions [54]. Interestingly, studies performed in mouse models documented that EMF treatment can reduce the growth and vascularization of breast cancer [55]. On the other hand, the EMF promotes bone healing by enhancing osteogenesis and vascularization of tissue-engineered constructs, especially when used in co-treatment with a vascular endothelial growth factor [56]. One may not exclude that this effect can be connected with the phenomenon of VCAM1-mediated proplatelet formation in the osteoblastic niche, which was previously documented in bone marrow [57]. Thus, there occurs a connection among EMF treatment, VCAM1 expression and action, and angiogenesis in a target tissue. Nevertheless, these conclusions, based on in vitro study, ought to be further validated in vivo.

The results of the current study indicate also that the EMF treatment increases *HOXD13* gene transcriptional activity, which enriches *positive regulation of biological process* GO BP term (GO: 0048518). The HOXD13 might be recognized as a cell proliferation promoter through binding to DNA replication origins and accelerating DNA synthesis [58]. The increased DNA synthesis and cell proliferation rate were demonstrated previously as the consequences of treatment with an EMF [59,60,61]. These observations suggest that there occurs a link among the EMF radiation, the increased transcriptional activity of the *HOXD13* gene, accelerated DNA synthesis rate, and increased rate of cell proliferation.

Interestingly, the altered expression of HOXD13 has been found in progesterone-positive breast cancer and thyroid tissue cancer [62,63,64]. Therefore, it is tempting to interpret the increased abundance of *HOXD13* mRNA observed in the myometrium in response to the EMF treatment as an indicator of neoplastic transformation in this tissue. Nevertheless, the results of the current study also demonstrated that EMF treatment causes a down-regulation of *cell division cycle 7* (*CDC7*) transcriptional activity. CDC7 is a kinase playing a crucial role in the initiation of DNA replication, and many cancer types and tumor cell lines are rather up-regulated [65,66,67,68]. Notably, although the radio frequencies of EMF (RF-EMF, 3 kHz–300 GHz) have indeed been defined by the International Agency of Research on Cancer (IARC) as probably carcinogenic, the novel reports indicate that ELF-EMF therapies might be helpful in the suppression of tumor growth and vascularization [69,70,71]. The endanger or beneficial health effects of exposition to an EMF might be strictly dependent on EMF frequency and duration of EMF treatment. Thus, the results of the current study do not deliver obvious premises to connect the exposition to the EMF at 50 Hz with the increased risk of neoplastic transformation in the myometrium. However, further in vivo studies are needed for understanding the relation among exposition to the EMF radiation and cancer development and progression.

Notably, this study documented that ELF-EMF treatment causes a down-regulation of the *EGR2* gene, enriching *positive regulation of biological processes* GO BP term. In women, elevated levels of *EGR2* mRNAs were identified in leiomyoma smooth muscle cells, whereas a knock-down of *EGR2* in these cells is associated with an increased cell proliferation rate [72]. Interestingly, treatment with the EMF caused also the down-regulation of the *retinol-binding protein* (*RBP4*) gene in the myometrium. RBP4, similarly to EGR2, was previously noted to impact cell proliferation rate, however, the knock-down of *RBP4* significantly decreases the proliferative potential [73]. Recently, it was documented that the EMF treatment affects cell proliferation rate, but the final result of this treatment, i.e., increase or decrease, was varying depending on cell type and used EMF parameters [74,75,76,77]. Taking into consideration results regarding the impact of the EMF treatment on the expression of *HOXD13, CDC7, EGR2,* and *RBP4*, the final result of this treatment can be the outcome of the alterations in the expression profile of genes and proteins involved in the regulation of the cell cycle or morphogenetic processes in affected cells.

Porcine myometrium during the peri-implantation period expresses epidermal growth factor (EGF) [78] and EGF receptor (EGFR) [18], and thus EGF-EGFR system might significantly contribute to the regulation of myometrial morphogenesis and cell proliferation. The results of the current study documented that there occurs a C > T substitution in 3’UTRvariant of EGFR. This alteration can affect the binding of the exact miRNA to the target sequence, leading to alterations in transcriptomic profile [79]. Although no significant alterations in EGFR expression were found in the myometrium treated with the EMF, the indicated C > T substitution in 3’UTRvariant of EGFR can cause a lesser potential of the myometrium to respond to signals triggered by EGF. Similarly, there was a predicted RNA editing region within PPP1R15B, encoding for protein phosphatase 1 regulatory subunit 15B. Specifically, there was found an A > G substitution in the intron variant of this gene despite not altered abundance of mRNA transcript. Notably, alterations in an intron or a downstream region of the gene of interest are believed to affect its expression. This notion was true for CIITA, in which ELF-EMF treatment-induced A > G substitution in downstream variants and an up-regulation of its transcriptional activity. Notably, the role of CIITA is inducing de novo transcription of major histocompatibility complex (MHC) class II genes and increasing constitutive MHC class I [80]. This phenomenon once again confirmed that short-lasting ELF-EMF treatment may contribute to the increase of immune responses in exposed tissues [38]; nevertheless, this assumption should be further confirmed using in vivo tests.

The results of the current study indicate that treatment with the EMF caused the alterations in the myometrial expression of genes enriching *secretion by cell* (GO:0032940), *peptide secretion* (GO:0002790), and *export from the cell* (GO:0140352) GO BPs, and *extracellular region* (GO:0005576) GO CC. Among DEGs, enriching these terms were the above described *RBP4* and *TNF* genes. Moreover, *extracellular region* term was enriched by down-regulated *PDYN*, *uteroferrin-associated basic protein-2* (*UABP-2*), and *hyaluronan and proteoglycan link protein 1* (*HAPLN1*). PDYN is a precursor for dynorphin synthesis [81], an opioid that contributes to the regulation of the HPA-axis and stress response mechanism [82], and is used in the treatment of pain [83]. Respecting that ELF-EMF therapies are used in the treatment of pain [41], the thinking is that the expression of *PDYN* mRNA in response to ELF-EMF treatment would be up-regulated. Nevertheless, the current study demonstrated that the EMF treatment significantly decreased myometrial synthesis of *PDYN* mRNA transcript. The expression of *PDYN* mRNA was previously found in the porcine endometrium [84]. It was determined that the expression of *PDYN* mRNA alters in response to IL 6, but only during days 10–11 of the estrous cycle [84]. During the peri-implantation period, none of the selected cytokines, i.e., IL 1*β*, IL 6, or TNF, affected endometrial *PDYN* mRNA transcript abundance [84]. It is not known yet whether pro-inflammatory cytokines may affect the myometrial expression of *PDYN* mRNA.

In conclusion, the EMF radiation induces alterations in transcriptomic profile in the myometrium of pigs during the peri-implantation period. Many of the evaluated DEGs in the consequence of EMF treatment encode for proteins involved in the regulation of immune responses which are essential for proper embryo-maternal communication during the fetal peri-implantation period. The observed transcriptomic alterations may affect the mechanisms of estrogens synthesis and release, and the process of angiogenesis in the myometrium, as well as may alter the proliferative potential, and/or neoplastic transformation of myocytes. Nevertheless, it should be emphasized that these results obtained with in vitro models should be further confirmed using in vivo tests. The observed EMF-related alterations in RNA edition sites and the expression of lncRNAs may be helpful to clarify the consequences of the EMF of extremely low frequency on the modulation of the transcriptomic profile and the activity of the myometrium.

## 4. Materials and Methods

### 4.1. Ethics Statement

Following the Act of 15 January 2015 on the Protection of Animals Used for Scientific or Educational Purposes and Directive 2010/63/EU of the European Parliament and the Council of 22 September 2010 on the protection of animals used for scientific purposes, the ethical review and approval were waived for this study, since all of the experiments were conducted on animal tissues collected post-mortem during regular economic slaughter provided in a professional slaughterhouse.

### 4.2. Animals, Collection of Myometrial Tissue, and EMF Treatment

All experimental procedures were performed using slices of the myometrium prepared within the previous study by Franczak et al. 2020 and Drzewiecka et al. 2021 [8,12]. Briefly, the myometrial tissue was obtained from pigs (*Sus scrofa domestica* L., Polish Landrace × Great White Polish, weighing 95–110 kg; *n* = 6) on days 15–16 of pregnancy. The myometrial slices (95–105 mg, 2–3 mm thick) were placed into 24-well culture plates and covered with preincubation medium (1 mL; M199, Sigma Aldrich, St Louis, MA, USA) supplemented with 0.1% of Bovine Serum Albumin (Carl Roth GmBH + Co KG, Mühl-burg, Karlsruhe, Germany) and 1% of antibiotic–antimycotic solution (Sigma Aldrich, St Louis, MA, USA), and preincubated for 2 h in a water-shaking bath at 37 °C in an atmosphere of 95% O_2_ and 5% CO_2_. Next, the preincubation medium was replaced with the fresh one of the same composition, and myometrial slices were incubated in vitro in Sham’s conditions (control group, *n* = 6), or were treated with the sinusoidal EMF at a frequency of 50 Hz (8 mT) for 2 h (experimental group, *n* = 6). During the incubation, the temperature was monitored to exclude the bias of the thermal effects. A detailed description of the EMF exposure system was presented in our previous study [8]. After incubation, slices of the myometrium were collected, washed in phosphate-buffered saline, dried, snap-frozen in liquid nitrogen (−196 °C), and stored at −80 °C for further transcriptome profiling.

### 4.3. RNA Isolation

Total RNA was extracted from myometrial slices following custom protocol using TRI Reagent (Sigma Aldrich, St. Louis, MA, USA) and RNeasy Mini Kit (Qiagen, Valencia, CA, USA). First, 20 µg myometrial fragments were minced thoroughly on ice in a diethyl pyrocarbonate-treated Eppendorf tube containing a 500 µL of TRI Reagent. Then, samples were homogenized with TissueRuptor homogenizer (Qiagen, Valencia, CA, USA) for 2 s, and mixed well by vortexing for 10 s. Next, samples were incubated on ice for 30 min, and briefly mixed by vortexing every 10 min of incubation until full digestion was completed. Next, 150 µL of ice-cold chloroform was added to each sample, mixed by pipetting, and incubated for the next 3 min. Samples were centrifuged 8000× *g*, 8 min, 4 °C in Centrifuge 5904R (Eppendorf, Hamburg, Germany). Afterward, the separated upper phase was transferred to another Eppendorf tube containing 200 µL of 70% ethanol, previously frosted to −20 °C, and mixed well by pipetting. Then, samples were transferred carefully on the RNeasy Mini spin column (Qiagen, Valencia, CA, USA), and after 1 minute of incubation centrifuged for 15 s, 8000× *g*, at room temperature. After binding RNA to the membranes of the columns, the RNA isolation was performed exactly as described in the Manufacturer’s protocol (Qiagen, Valencia, CA, USA). RNA was eluted with 80 µL of DEPC-treated water and instantly measured spectrophotometrically for an initial determination of the purity (optical density, OD, A260/A280) and concentration (ng/µL) of obtained RNA. Only samples with OD 1.8–2.0 with concentration > 500 ng/µL proceeded further. Next, aliquots of the obtained RNA were prepared for further measurement of RNA integrity number (RIN; 28 S/18 S ratio) using 2100 Bioanalyzer with RNA 6000 Nano LabChip kit (Agilent Technologies, Santa Clara, CA, USA), next-generation sequencing (NGS) (Illumina Macrogen, Seoul, North Korea), and the validation of NGS results analyses. Prepared aliquots were frozen at −80 °C, and each aliquot was handled only once through the freezing-refreezing cycle.

### 4.4. Construction and Sequencing of cDNA Libraries—Next-Generation Sequencing

The construction and sequencing of cDNA libraries was performed for *n* = 3. The number of animals per group (*n* = 3) was selected due to the results of power analysis performed with the use of RNASeqPower (v. 1.32.0) Bioconductor (v. 3.13) library. The theoretical calculation assumed to obtain an average of 50 mln raw paired-end reads per sample with a coefficient of variation of expressed data equaling 0.1 (applied in inbreed animal objects), and minimal fold change (FC) of expression equaling to 2. RNA aliquots that exhibited RIN ≥ 8, rRNA ratio > 1.0, concentration > 20 ng/µL, total content > 1 µg, total volume > 50 µL were used for the construction and sequencing of cDNA libraries. Both the construction and sequencing of cDNA libraries were held by an outsourcing company (Macrogen, Seoul, North Korea) operating on Illumina NovaSeq 6000 System (Illumina, San Diego, CA, USA). Briefly, there were constructed TruSeq mRNA stranded libraries, and the sequencing was performed selecting the following run configurations: 2 × 150 bp and throughput 40 M paired reads per sample. The raw data were submitted to the European Nucleotide Archive (ENA) under accession No. PRJEB42848, for further transcriptome profiling and bioinformatic analysis of gene expression.

### 4.5. Transcriptome Profiling

Transcriptome profiling was performed for samples of the control and the experimental RNA-seq libraries. Digital sequences of paired-end reads were saved in FASTQ format and evaluated with FASTQC software (v. 0.11.9, Babraham Institute, Cambridge, Great Britain) [85]. The quality control was conducted with the use of Trimmomatic software (v. 0.38) [86], and the Illumina adaptors and low-quality reads (with PHRED cutoff score ≤ 20 and 10 bp frameshift region) were removed. The remaining paired-end reads were aligned to the pig reference genome with ENSEMBL annotation (Sus_scrofa.Sscrofa11.1.98) using the Spliced Transcripts Alignment to a Reference (STAR) mapper (v.2.7.3, Cold Spring Harbor Laboratory, New York, NY, USA) [87] and StringTie (v. 1.3.3, Baltimore, MD, USA) [88] pipeline. Count values obtained within STAR method (quantMode GeneCounts) were assigned to the TARs. Porcine TARs were divided into protein-coding genes and uncovered regulatory region groups. Differential expression of genes was estimated with two statistical methods: DESeq2 (v. 1.32.0) [89] and edgeR (v. 3.34.0) [90] in R (v. 4.1.0)/Bioconductor software [91]. Only transcripts that exhibited an adjusted *p*-value < 0.05 and an absolute value of logarithmic Fold Change (logFC) > 1 determined by both indicated methods were defined further as the DE-TARs. The DE-TARs were divided into DEGs and DE-ncRNAs.

Next, the evaluated DEGs were classified to GO terms and biological pathways using gProfiler software (v. e98) [92], the Kyoto Encyclopedia of Genes and Genomes (KEGG, v. 96) [93], and REACTOME (v. 75) databases [94]. The enrichment classification was conducted at the adjusted *p*-value < 0.05. The final consensus DEGs were visualized in an MA, a Volcano, and circos-heatmap plots with gplots (v. 3.1.1), circlize (v. 0.4.13) Bioconductor package, and a custom script in R.

Additionally, using the multi-stage long noncoding (lnc) RNA identification procedure DE-ncRNAs were divided into DE-lncRNAs, and other unclassified DE-ncRNAs. The pool of DE-lncRNAs included transcripts with ENSEMBL lncRNA biotype and uncovered transcripts with length > 200 bp; multi-exonic structure and no protein-coding potential. The coding potential was tested by three algorithms: by the Coding-Potential Assessment Tool (CPAT; v. 1.2.2, Beijing, China) [95], Coding Potential Calculator (CPC; v. 2, Beijing, China) [96], and predictor of long non-coding RNAs and messenger RNAs based on an improved k-mer scheme (PLEK; v. 1.2, Xidian University, Xi’an, China) [97] with default parameters. The expression correlations trans-acting links between DE-lncRNAs and mRNAs was investigated using a custom R script. Trans-acting relationships were determined based on Pearson’s correlation coefficient (r > 0.9 or r < −0.9) calculation of expression profiles.

### 4.6. RNA Editing Sites Prediction

For discovering the potential RNA editing sites with differences in the allele fraction between the EMF-treated and control samples, the mapped RNA-seq libraries were saved in Binary Alignment Map (BAM) files were recalibrated by Picard tool (v. 2.1.1, Broad Institute of MIT and Harvard, Cambridge, MA, USA) (http://broadinstitute.github.io/picard, accessed 18 February 2021). Next, variant calling analysis was performed, and the SNVs were retrieved using the golden-standard GATK (v. 3.6, Broad Institute of MIT and Harvard, Cambridge, MA, USA) [98]. The reference and alternative allele frequencies (AAF) of the identified SNVs were calculated and compared with the python rMATS-DVR scripts (University of California, Los Angeles, CA, USA) [99]. Using ENSEMBL VCF and GTF files, the SNPs annotation and gene location were assigned to the expressed variants. Obtained SNVs were filtered out according to the GATK standard parameters: the depth coverage < 10; RMSMappingQuality < 40; QualitybyDepth < 2; MappingQualityRankSum < −12.5; and ReadPosRankSum < −8. High-quality SNVs with alternative allele occurrence in at least half RNA-seq samples were conducted for the downstream analysis. Subsequently, SNVs located in the vicinity of the spliced junction sites within bidirectional genes and pseudogenes were eliminated from the identification of the RNA editing variations. Filtrated potential RNA editing candidates with no SNP annotation (rs ID) were divided into canonical (A-to-I and C-to-U) and noncanonical (all other possible base substitutions) substitutions. The AAF changes (∆AAF > 0.1; FDR < 0.001) between the EMF-treated and controls were evaluated only for canonical RNA editing substitutions. Additionally, an allelic imbalance ratio was tested by the chi-square goodness-of-fit method using the R environment. All RNA editing candidates were annotated by variant effect predictor (VEP, v.98, EMBL-EBI and Wellcome Trust Sanger Institute, Oxford, Great Britain) [100] and were plotted by Circos (v. 0.69-9, Canada’s Michael Smith Genome Sciences Centre, Vancouver, BC, Canada) [101].

### 4.7. Validation Procedure

The validation procedure was performed by testing the relative mRNA transcript abundance (Real-Time PCR) of seven selected DEGs in the myometrial tissue treated in vitro with the EMF at 50 Hz (*n* = 6) when compared to control (no EMF-treated, *n* = 6). The selection of DEGs for validation was grounded on their FC values and contribution to evaluated GO terms. These were *HOXD13*, *PDYN*, *VCAM1*, *IL15*, *STAT5A*, *TNF*, and *EGR2*. The amplification of selected DEGs mRNA was conducted using TaqMan™ RNA-to-CT™ 1-Step Kit and specific, pre-designed TaqMan™ probes presented in Table 2 (Both Thermofisher Scientific, Waltham, MA, USA). The amplification was conducted in an AriaMX system (Agilent Technologies, Santa Clara, CA, USA) in 10 µL of total reaction volume using a 4 pg/µL of total RNA. The analysis was conducted following the general guidelines for gene expression analysis [102], and standard manufacturer’s protocol. The relative mRNA transcript abundance was calculated using the ΔΔCt method, selecting beta-actin (ACTB) and glyceraldehyde 3-phosphate dehydrogenase (GAPDH) as reference genes.

## Figures and Tables

**Table 1:** GO terms and KEGG and REACTOME pathways evaluated in the myometrium treated with an EMF at 50 Hz for 2 h.

Term Name	Term ID	Adjusted *p*-Value
GO BP		
Defense response	GO:0006952	0.00002344438413677965
Immune response	GO:0006955	0.0031852702186542744
Immune system process	GO:0002376	0.005703042047974045
Positive regulation of biological process	GO:0048518	0.009437770426041985
Defense response to other organism	GO:0098542	0.015461210039798225
Secretion by cell	GO:0032940	0.017106824946529414
Immune effector process	GO:0002252	0.0175096188187713
Peptide secretion	GO:0002790	0.018160684956297413
Response to other organism	GO:0051707	0.020710132468959617
Response to external biotic stimulus	GO:0043207	0.02169456662389253
Export from cell	GO:0140352	0.025696623178163416
Response to biotic stimulus	GO:0009607	0.027680534899551905
GO CC		
Extracellular region	GO:0005576	0.009251891504629992
KEGG pathways		
Rheumatoid arthritis	KEGG:05323	0.0025090688431046978
TNF signaling pathway	KEGG:04668	0.008835243157774076
African trypanosomiasis	KEGG:05143	0.03556479600406204
REACTOME		
Regulation of IFNA signaling	REAC: R-SSC-912694	0.005030993232569516
Interferon-alpha/beta signaling	REAC: R-SSC-909733	0.03000607786831512

**Table 2:** Taq Man probes used in the validation of the NGS results experiment.

Fold Change ^1^	Gene Symbol	Assay ID
Differentially expressed genes		
7.339974418	*HOXD13*	Ss06916655_m1
7.084714455	*PDYN*	Ss03391715_m1
4.690302179	*VCAM1*	Ss03390912_m1
3.659258776	*IL15*	Ss03394854_m1
2.971483779	*STAT5A*	Ss03394621_m1
2.81226065	*TNF*	Ss03391318_g1
−1.878442068	*EGR2*	Ss03388929_m1
Reference genes		
-	*ACTB*	Ss03374854_g1
-	*GAPDH*	Ss03376081_u1

^1^ Fold change (*p* ≤ 0.05) obtained after transcriptome profiling within NGS analysis.

## Data Availability

All of the data are presented in the study. The raw data used for the preparation of the presented results are available in the European Nucleotide Archive (ENA) under accession No. PRJEB42848.

## Supplementary Materials

The following are available online at https://www.mdpi.com/article/10.3390/ijms22147322/s1, Figure S1: (A) PCA plot. Sports represent biological report (n) for control group (red, sports, no EMF-treated) or experimental group (blue sports, EMF-treated). (B) Hierarchical clustering of RNA-seq results, Table S1: A full list of evaluated differentially expressed TARs in response to ELF-EMF (50 Hz, 8 mT, 2 h) in vitro treatment in the myometrium of the pig during the peri-implantation period, Table S2. A full list of evaluated GO terms, KEGG and REACTOME pathways to which were assigned differentially expressed transcription active regions in response to EMF (50 Hz, 8 mT, 2 h) in vitro treatment in the myometrium of the pig, Table S3. The predicted RNA editing sites in ELF-EMF treated (50 Hz, 8 mT, 2 h) myometrial tissue of the pig during the peri-implantation period.
