# Supplementary figures and images for: Effect of the Electromagnetic Field (EMF) Radiation on Transcriptomic Profile of Pig Myometrium during the Peri-Implantation Period—An In Vitro Study
Source: Int J Mol Sci. 2021 Jul 7;22(14):7322. doi: 10.3390/ijms22147322 (PMC8305477; doi:10.3390/ijms22147322)

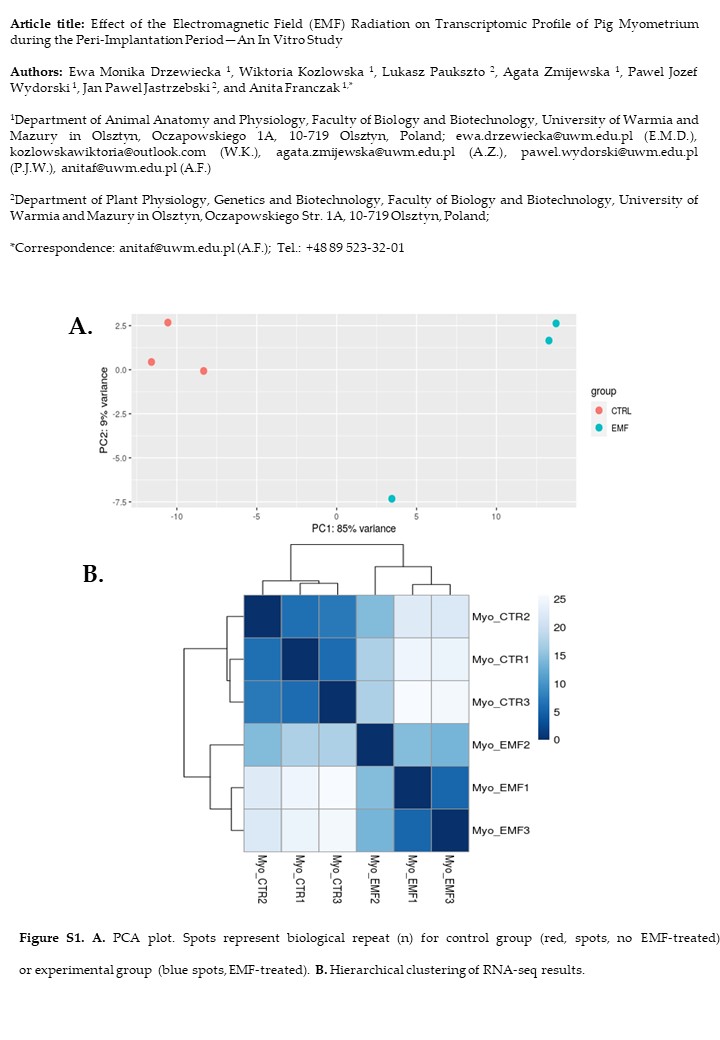

Supplement: Supplementary file 1 [file ijms-22-07322-s001.zip › Supplementry files/Figure S1.jpg]
